# Remote sensing-based measurement of Living Environment Deprivation: Improving classical approaches with machine learning

**DOI:** 10.1371/journal.pone.0176684

**Published:** 2017-05-02

**Authors:** Daniel Arribas-Bel, Jorge E. Patino, Juan C. Duque

**Affiliations:** 1 Department of Geography & Planning, University of Liverpool, Liverpool, United Kingdom; 2 Research in Spatial Economics (RiSE-group), Department of Economics, Universidad EAFIT, Medellín, Colombia; University of Vermont, UNITED STATES

## Abstract

This paper provides evidence on the usefulness of very high spatial resolution (VHR) imagery in gathering socioeconomic information in urban settlements. We use land cover, spectral, structure and texture features extracted from a Google Earth image of Liverpool (UK) to evaluate their potential to predict Living Environment Deprivation at a small statistical area level. We also contribute to the methodological literature on the estimation of socioeconomic indices with remote-sensing data by introducing elements from modern machine learning. In addition to classical approaches such as Ordinary Least Squares (OLS) regression and a spatial lag model, we explore the potential of the Gradient Boost Regressor and Random Forests to improve predictive performance and accuracy. In addition to novel predicting methods, we also introduce tools for model interpretation and evaluation such as feature importance and partial dependence plots, or cross-validation. Our results show that Random Forest proved to be the best model with an *R*^2^ of around 0.54, followed by Gradient Boost Regressor with 0.5. Both the spatial lag model and the OLS fall behind with significantly lower performances of 0.43 and 0.3, respectively.

## Introduction

The use of remote sensing data to gather socioeconomic information is based on the premise that the physical appearance of a human settlement is a reflection of the society that created it and on the assumption that people living in urban areas with similar physical housing conditions have similar social and demographic characteristics [1, 2]. The number of studies that address the usefulness of remote sensing imagery to estimate socioeconomic variables has increased in recent years [3–9]. This trend is related to the increasing availability of commercial satellite platforms and the decreasing costs of this kind of data [10, 11]. Remote sensing imagery could be used as an alternative source of information in urban settings when survey data is scarce or to update socioeconomic data for different dates than those of surveys or censuses [7].

Relationships between socioeconomic variables and remote sensing-derived variables have been quantified in different cities around the world [9, 12–15]; however, local research is always needed, as the particular conditions of each city must be taken into account to find useful correlations [16], and findings for one city can be quite the opposite for another city, even in the same country; e.g., the amount of vegetation is an indicator of urban decay in Detroit, Michigan, while the same variable is positively correlated with income, house value and educational attainment in Denver, Colorado, and Phoenix, Arizona, among others [10, 17]. Accordingly, the image-derived variables that best relate to a specific socioeconomic index can change among different cities, but it is important to unveil those relationships to help identify patterns that could be useful in different settings.

One of the most recurrent applications in this field is the measurement of poverty in urban areas. According to [11] at least 87 works on this topic have been published in the last 15 years, most of them focused on slum mapping or slum detection [18–21]. However, only a limited number of works have focused on the usefulness of remote sensing imagery to quantitatively estimate indices of urban poverty. [22] used reflectance data from Landsat imagery to estimate a residential quality index in Sydney, Australia. [23] and [24] used land cover descriptors and texture measures from medium to very high spatial resolution satellite imagery for predicting the Slum Index as a function of remote sensing-derived variables in Accra, Ghana. [25] related the percentage of areas with non-formal building standards, identified from Ikonos imagery, with an Index of Multiple Deprivation at the ward level in Delhi. More recently, [7] used data drawn from an RGB composition of a Quickbird scene with a spatial resolution of 0.60 m to estimate the Slum index in Medellin, Colombia.

The quantification of an index over the whole area of a city, covering the most deprived areas to the most affluent, would better inform urban planning policies than the mere identification of slum pockets. According to [26], the repeat cycle and wide area coverage of remotely sensed data provide a mean for monitoring urban processes to help in the analysis and response by policy makers. If an approach to quantifying an index of urban quality based on remote sensing is demonstrated to be possible and repeatable, it could be useful to identify urban places that show a trend of decreasing quality and take action before they reach critical conditions.

In this paper, we use the Living Environment Deprivation (LED) index from the English Indices of Deprivation for Liverpool Statistical Areas to explore the potential benefits of two Machine Learning methods, Gradient Boost Regressor (GBR) and Random Forest (RF), that are used to predict the LED index based on data entirely derived from images. We compare the results with the outcomes of two classic econometric models –Ordinary Least Squares regression (OLS) and a Spatial Lag model (SL) based on the generalized method of moments. According to [27], there are two broad domains of neighborhood attributes that may be relevant to public health: features of the physical environment and features of the social environment. Housing conditions and the surrounding environment characteristics have been found to be associated with health status and overall quality of life [28]. Remote sensing data has already been used to quantify urban quality of life indexes [22, 29–31]. We chose the LED index because it measures the quality of the local environment, it is related to urban quality of life concepts, and it is, at least partially, based on some aspects of the dwellings that are reflected in the appearance of the urban settlement. The LED index is a suitable tool for the characterization of the urban physical environment [32]. We use four sets of variables extracted from a very high spatial resolution (VHR) image downloaded from Google Earth (GE): a set of land cover features, a set of spectral features, a set of texture features, and a set of structure features.

Most previous works on the relationship between remote sensing-derived variables and deprivation indices use expensive commercial imagery with several spectral bands in the visible and near-infrared fractions of the electromagnetic spectrum [11, 33] as well as three-dimensional data such as LIDAR [34]. However, many cities and local governments in both the developed and developing countries do not have the resources to purchase full satellite imagery or LIDAR data and restrict themselves to using RGB data for data extraction via interpretation [35]. Google Earth (GE) imagery may be the only available source of aerial imagery for small local governments. GE images are accessible to the public, albeit bound by their terms of use, and their ability to provide useful information for policy making is worth testing [36, 37]. Works similar to this have tested the relationship of image-derived variables with poverty indices in cities of developing countries within the Tropical Zone (Accra, Ghana, and Medellin, Colombia). Liverpool is an interesting case study because it is located in a developed country but is home to some of the most deprived areas in the United Kingdom.

Methodologically, previous works focusing on the estimation of poverty indices from remote sensing data used classic econometric approaches like correlation analysis, linear regressions, and spatially adjusted regressions [7, 23–25]. Recent literature contributions have explored the potential of machine learning to identify unstructured human settlements using remotely sensed imagery. The applied techniques include neural networks [5, 38], random forest [6, 39], and support vector machine [39]. The main advantages of these approaches are their usefulness in areas with poor survey coverage, their good prediction power and, as stressed by [6], some versions of these models are capable of dealing with the non-linear relationships between poverty and environment.

With this paper we expect to contribute in two key aspects: First, we want to explore the usefulness of a new and wide set of image-derived variables (spectral, texture and structure features) that can be easily estimated and that can be a good complement of other most common variables such as land cover types. In this context, machine learning techniques give us the possibility of including any number of variables without being concerned about multicollinearity as well as to capture non-linear relationships. If successful, the approach tested here could be standardized and automated given its flexibility and ease of implementation. Hence, it could be useful to help monitor quality of life in cities over time and to quantify the changes in its spatial pattern across the city between different dates even in cases with a limited budget. Second, by using Liverpool as case study, we want to contribute new empirical evidence on the understanding of some quantifiable aspects of the appareance of cities in developed countries that relate to urban quality of life and other similar indices. Other comparable researches have focused on North American cities [40–43]. The most important findings in these studies are the high explanatory power of the presence of impervious surfaces (computed as the percent of impervious surface cover) and the presence of vegetation (quantifyed as the percent of vegetation cover, or the mean Normalized Difference Vegetation Index, NDVI); and the evidence of spatial instability of these relationships, which requires the use of spatial econometric techniques such as the Geographically Weighted Regression.

The remainder of the paper is organized as follows. Section presents the study area, the LED index, and the variables derived from the VHR image. Section 0.2 describes the two classic econometric models and the two machine learning methods compared in this study. The results and subsequent discussion are presented in sections 0.6 and 0.8. Finally, section 0.8 presents the main conclusions.

## Data

**Fig 1:**
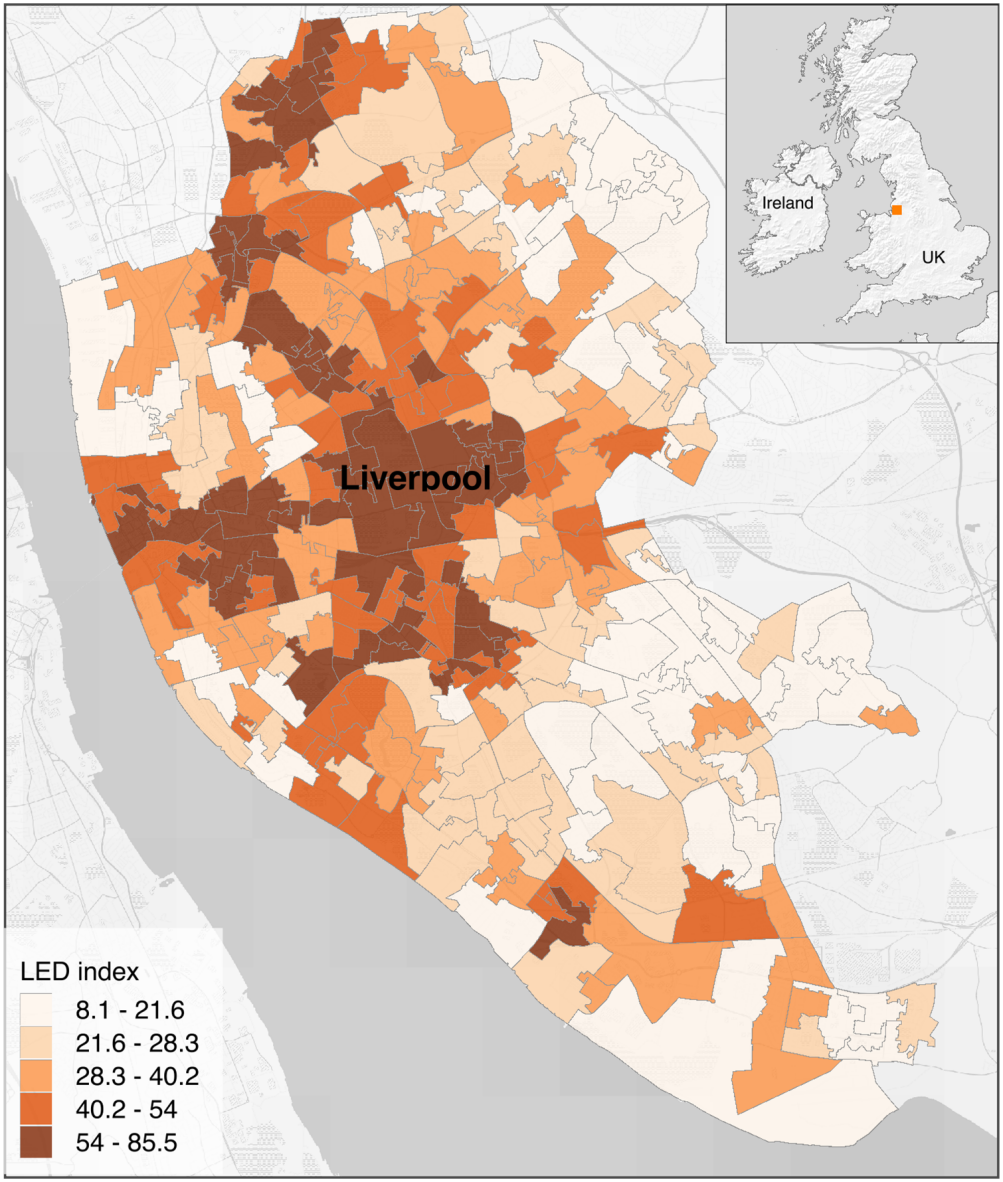
Location of Liverpool city and LED index values at LSOA level, shown over a Stamen Terrain base map (base map tiles by Stamen Design, under a CC BY 3.0, data by OpenStreetMap, under CC BY SA).

Our analysis is geographically focused on the British city of Liverpool. Liverpool is located in the North West of England (see Fig 1) and is among its ten largest cities. Its exact position varies depending on the city definition used. For example, if the built-up area definition is chosen, Liverpool is the sixth largest in the UK [44]. As of 2015, the Office of National Statistics estimated just under 480,000 people were living within the boundary of the local authority. Liverpool has a historical trajectory that shapes its spatial structure and defines the way the city looks today. According to the city profile by [45], “[it] pioneered many of the elements of the modern industrial metropolis, only to deurbanise during a ruinous late 20th century decline, halving its population.” The combination of such a changing and uneven landscape with its Western European location make Liverpool a good candidate for our exercise. On the one hand, we expect several historical layers of development, rise, and decline to be reflected in its spatial structure and, hence, in the way the city looks from the sky. On the other hand, its location in a developed country with a temperate latitude provides a very different testbed than that usually featured in other studies of remote sensing-based measurement of socio-economic characteristics.

Within Liverpool, we use the Lower Layer Super Output (or LSOA in its most common acronym; [46]) geography. LSOA affords us spatial resolution while allowing us to connect our satellite-derived measures with many additional datasets. Crucially, we are interested in the Index of Multiple Deprivation (IMD, [32]), produced by the Department for Local Communities and Local Government. Within the IMD, we focus on the component that relates most to the appearance of the landscape and, hence, is expected to be best captured by satellite imagery, i.e., living environment.

### 0.1 Living Environment Deprivation

The LED index is one of the seven dimensions that compose the English Indices of Deprivation, which includes income; employment; health and disability; education, skills and training; crime; barriers to housing and services; and living environment. According to [32], the model of multiple deprivation is based on the idea that (1) the separate dimensions of deprivation can be recognized and measured separately, (2) these dimensions of deprivation are experienced by individuals living in an area, and (3) an area-level measure of deprivation for each of the dimensions can be calculated [32]. These indices are reported at the LSOA level for the entire country and they allow the comparison of deprivation in one area relative to other areas, but they cannot be treated as absolute measures of deprivation for each place.

The LED index measures the quality of the local environment in terms of the quality of housing and the quality of the surrounding environment, and it is composed of four indicators: housing in poor condition, houses without central heating, outdoor air quality, and road traffic accidents [32]. The quality of the urban environment is an issue of fundamental concern for citizens, researchers, and policy makers [47, 48]. Urban green spaces are linked to a range of health metrics: urban vegetation provide benefits to people by improving air quality (oxygen production and carbon sequestration), abatement of noise pollution, mitigation of the urban heat island effect, and reduction of storm-water [49, 50]. Previous works have related urban environmental problems with public health outcomes [27, 51]. Urban green spaces measures, like the share of vegetation within an area and the distance to the closest green patch, are often used in studies examining people’s physical health and behavior [52]. Lack of access to parks and green spaces have been linked to mortality, higher rates of obesity, and mental issues [53]. According to [54], remotely sensed data can be used to detect and measure urban changes and provide data for the analysis of the impacts of urbanization on the environment and human health. We chose to test the relation of the LED index with LSOA descriptors extracted from VHR imagery because this index is partially based on physical aspects of the dwelling units that can be reflected in the appearance of the urban layout. Fig 1 shows the LED index in Liverpool at the LSOA level, classified in 5 intervals using the Quantile method. Values range from 8.1 to 85.5, and the map shows a clear spatial pattern with higher values around the center of the city and toward the north and west and lower values in the periphery.

### 0.2 Remote sensing derived variables

We downloaded the most updated (up to February 2016) GE images of Liverpool city with enough zoom level to be similar to VHR imagery with sub-meter pixel size using a tool for automatic downloading of image tiles from the Google Satellite Map web service (http://www.allallsoft.com/gsmd); we then combined them into a single mosaic in geographic coordinates. According to the data provider information, displayed in Google Earth, and the Google Earth blog, these Liverpool VHR imagery were collected by Google itself in 4th April 2015. We projected the image mosaic onto the same coordinate system of the spatial database of deprivation indices of Liverpool and obtained a georeferenced image with a pixel size of 70 cm. We used that image as an input to calculate image-derived features or variables for the same spatial units for which the LED index is reported (LSOA).

Previous studies have related intra-urban measures of deprivation with different types of image-derived variables. The presence of impervious surfaces, the amount of vegetation, the amount of bare soil, and the share of orange clay roofs within an area have been related to intra-urban poverty indices in previous works [7, 23, 24]. Some texture measures [23, 51] and spatial pattern descriptors [7, 51] have been tested as well. Moreover, several image texture measures have been demonstrated to be useful for differentiating slum settlements from formal settlements in several cities of developing countries around the world [20, 21, 55]. Vegetation proxies derived from remote sensing, like the mean NDVI, the Soil Adjusted Vegetation Index (SAVI), the Leaf Area Index (LAI), or the fraction of vegetation cover within an area, have been related to income and other socioeconomic variables in several American cities [12, 13, 29, 40, 56, 57].

A per-pixel classification of the scene is performed to calculate a set of land-cover variables within LSOA polygons. Next, we used an automatic tool for image feature extraction at the object level called FETEX 2.0 [58] to extract spectral, texture and structure features at the LSOA level. FETEX, developed at the Geo-Environmental Cartography and Remote Sensing Research Group (http://cgat.webs.upv.es), is a computer package for image, object-oriented feature extraction [58]. We calculated three sets of variables using FETEX and the boundaries of LSOA: a set of spectral features, a set of texture features and a set of structural features. Spectral features convey information about color in each image RGB band, while texture and structure features inform about the spatial arrangement of the elements within the image using the intensity values of the red band.

**Land cover features**
Land cover features describe the composition of the urban scene in terms of the amount of basic land cover types: vegetation, soil, gray impervious surfaces (asphalt and industrial roofing), orange impervious surfaces (clay tile roofs and similar), shadow and water. The composition of the urban landscape has been related to urban quality of life in previous works [22, 30, 31, 41, 56, 57]. Urban green spaces are considered as an important urban amenity to improve air quality and to promote citizen’s physical activity [53, 59]. In general, urban areas with a high share of vegetation coverage are expected to be associated with low values of the LED index; while areas exhibiting a high fraction of (bare) soil coverage are expected to be associated with higher values of the LED index. We used the red, green and blue bands of the GE image as inputs to a supervised per-pixel classification in ENVI software to differentiate these land cover classes using a maximum likelihood classifier. We decided to use this classic method to maintain the image processing flow as less sophisticated as possible, to introduce the machine learning algorithms only in the analysis of the relationships of the LED index with the image-derived variables. The classification accuracy was assessed using a point-based technique with a reference dataset of randomly selected points. We collected a sample of on-screen ground truth points and divided it randomly into two datasets: 80% as the classification training set and 20% as the validation set. Table 1 shows the confusion matrix as well as the overall accuracy and the kappa coefficient of the classification results.
  The classified image at the pixel level was used to calculate the following aggregated land cover variables at the LSOA level: the percentage of impervious surfaces (gray and orange), the percentage of orange impervious surfaces, the percentage of impervious surfaces without orange surfaces, the fraction of orange surfaces over the impervious surfaces, the percentage of vegetation, the percentage of soil, the percentage of shadows, and the percentage of water.

**Table 1:** Accuracy assessment of GE image classification results.

Ground truth	Gray imp. surf.	Soil	Orange imp. surf.	Vegetation	Shadow	Water	Total
Classified as:							
Gray imp. surf	5,241	73	278	80	32	0	5,884
Soil	422	740	200	15	0	0	1,377
Orange imp. surf.	65	6	1,696	17	0	0	1,784
Vegetation	156	10	49	3,684	168	0	4,067
Shadow	1,474	6	68	496	1,427	0	3,471
Water	12	0	0	208	34	885	1,139
Total	7,550	835	2,291	4,500	1,661	885	17,722
Producer’s accuracy (%)	71.80	88.62	74.03	81.87	85.91	100	
User’s accuracy (%)	92.13	53.74	95.07	90.58	41.11	77.70	
Overall classification accuracy (%)		78.17					
Kappa value		0.71

**Spectral features**
The spectral features are the summary statistics of pixel values inside objects; i.e., LSOA boundaries in this study case. These features inform about the spectral response of objects, which depends on land coverage types, state of vegetation, soil composition, building materials, etc. [58]. These features complement the information provided by the land cover features, as they give a general measure of the color of the areas. For example, we expected high LED index values to be more common in the areas with few vegetated spaces, which usually present low mean spectral values in the green band. We selected the mean and standard deviation in each RGB band for this exercise. These features are easy to understand and could convey more information about the spectral differences across the small areas of the city than the other summary statistics for the intensity values of the image (minimum, maximum, majority, range, and sum).

**Texture features**
Texture features characterize the spatial distribution of intensity values in the image and provide information about contrast, uniformity, rugosity, etc. [58]. Texture measures have been related to an inhabitability index in Recife and Campinas, Brazil [60] and to identify urban slums in Hyderabad, India [21], and Casablanca, Morocco [20]. The kurtosis and skewness features are based on the histogram of the pixel values inside each LSOA polygon. Classic image texture features, proposed by [61], are computed from the Grey Level Co-occurrence Matrix (GLCM). The GLCM describes the co-occurrences of the pixel values that are separated at a distance of one pixel inside the polygon, and it is calculated considering the average value of four principal orientations, 0°, 45°, 90° and 135°, to avoid the influence of the orientation of the elements inside the polygon [58]. These texture variables include uniformity, entropy, contrast, inverse difference moment, covariance, variance, and correlation. The edgeness factor is another useful feature that represents the density of edges present in a neighborhood, and the mean and standard deviation of the edgeness factor are also computed within this set of texture features in FETEX 2.0 [58]. We expect that texture features help in the estimation of LED index values by accounting for some aspects of the distribution of the elements within the urban fabric. As an example, areas with high LED values are expected to be associated with high values of mean edge density and entropy. This is because these features account for the presence of different elements within the urban layout and this situation is more common in the areas that show small dwelling units and with few homogeneous vegetated spaces or water bodies.

**Structure features**
Structure features provide information on the spatial arrangement of elements inside the polygons in terms of randomness or regularity of the distribution of the elements [58, 62, 63]. Structure feature values indicative of high local heterogeneity and low regularity have been associated with deprived urban areas in some countries [7]. We would expect that these set of features could be useful to improve the image-based estimations of the LED index for Liverpool. For the current analysis, we computed a set of structure features at the LSOA level using FETEX 2.0 and the experimental semivariogram approach. According to [58], the semivariogram quantifies the spatial associations of the values of a variable, measures the degree of spatial correlation between different pixels in an image, and is a suitable tool for the characterization of regular patterns. FETEX 2.0 generates the experimental semivariogram of each polygon by computing the mean of the semivariogram calculated in six different directions, from 0° to 150°, with step increments of 30°. Then, each semivariogram curve is smoothed using a Gaussian filter to reduce measurement error [58]. Structure features extracted from the semivariogram are based on the zonal analysis defined by a set of singular points on the semivariogram, such as the first maximum, the first minimum, and the second maximum [58, 62, 63]. Table 2 shows the complete list of remote sensing variables used in our analysis.

**Table 2:** Remote sensing derived variables and descriptions.

Group	Variable name	Description
Land cover	p_imp_surf	Percentage of impervious surface cover
	p_ora_surf	Percentage of orange impervious surface cover
	p_i_s_wora	Percentage of impervious surface without orange surface cover
	f_or_imp_s	Fraction of orange impervious surface over the impervious surface
	p_veg	Percentage of vegetation cover
	p_soil	Percentage of bare soil cover
	p_shadow	Percentage of shadow cover
	p_b_water	Percentage of water cover
Spectral	MEAN1	Mean of band 1 intensity values (red color)
	DEVST1	Standard deviation of band 1 intensity values
	MEAN2	Mean of band 2 intensity values (green color)
	DEVST2	Standard deviation of band 2 intensity values
	MEAN3	Mean of band 3 intensity values (blue color)
	DEVST3	Standard deviation of band 3 intensity values
Texture	MEAN_EDG	Mean of the edgeness factor
	STDEV_EDG	Standard deviation of the edgeness factor
	UNIFOR	GLCM uniformity
	ENTROP	GLCM entropy
	CONTRAS	GLCM contrast
	IDM	GLCM inverse difference moment
	COVAR	GLCM covariance
	VARIAN	GLCM variance
	CORRELAC	GLCM correlation
	SKEWNESS	Skewness value of the histogram
	KURTOSIS	Kurtosis value of the histogram
Structure	RVF	Ratio variance at first lag
	RSF	Ratio between semivariance values at second and first lag
	FDO	First derivative near the origin
	SDT	Second derivative at third lag
	MFM	Mean of the semivariogram values up to the first maximum
	VMF	Variance of the semivariogram values up to the first maximum
	DMF	Difference between the mean of the semivariogram values up to the first maximum and the semivariance at first lag
	RMM	Ratio between the semivariance at first local maximum and the mean semivariogram values up to this maximum
	SDF	Second order difference between first lag and first maximum
	AFM	Area between the semivariogram value in the firs lag and the semivariogram function until the first maximum

## Methods

The derived features are not particularly useful for explaining LED by themselves. They need to be combined into a single model that creates predictions based on existing estimates. Conceptually, this may be represented as:

$$\begin{array}{c} LED=f(LC,SP,TX,ST) \end{array}$$ (1)

where *f*(⋅) is a function that combines information on land cover (*LC*), spectral (*SP*), texture (*TX*), and structure (*ST*) from each area to produce a prediction of its LED index. This presents one of the main methodological contributions of this paper. Following the existing literature on the topic, we use standard methods such as (spatial) regression to produce a linear combination of several features into an estimate of the LED index. Unlike most studies in the literature, however, we extend traditional linear models and adopt a more modern approach of prediction using machine learning techniques such as random forests and gradient boosting regressions. The remainder of the section offers an overview of each of the approaches we use to estimate Eq (1), and their relative advantages and disadvantages are addressed.

### 0.3 Baseline linear model

Our first approach, which serves as a benchmark, is to assume a linear combination to approximate *f*(⋅). Mathematically, this implies that Eq (1) becomes:

$$\begin{array}{c} LED=\alpha +\beta LC+\gamma SP+\delta TX+\zeta ST+\epsilon \end{array}$$ (2)

where *α*, *β*, *γ*, *δ* and *ζ* are (vectors of) parameters and *ϵ* is an error term assumed to be i.i.d. following a Gaussian distribution.

This approach is simple to implement as it is straightforward to obtain estimates of the parameters using Ordinary Least Squares (OLS); it has also been proven to be robust by a large number of studies in the literature. It is also relatively easy to interpret: Because the units in which the variables are expressed are known, the estimated coefficients provide information about the marginal change in the outcome associated with a unit increase in each of the predictors. However, its simplicity is also its most limiting factor. In a context where the priority is to obtain good predictions, a linear model can be too simple. The predictive performance of linear models in cases where relationships are more complex (e.g., nonlinear) is usually rather limited. Additionally, the technical mechanism used by OLS to compute estimates implies that it is sensitive to multicollinearity and that sometimes it is necessary to drop some variables from the set of predictors and thus potentially miss useful information at hand.

### 0.4 Spatial linear model

One way to improve the predictive performance of a linear model, while maintaining much of its interpretability, may be to extend it to accommodate spatial autocorrelation. In cases where the spatial nature of the data is relevant to the process being studied, formally including space in the statistical framework used is likely to improve the accuracy of its predictions. In our case, it is reasonable to expect that due to the inherent spatial variation of the LED index and to data issues such as the modifiable areal unit problem [64, MAUP], an explicitly spatial approach can prove beneficial.

In the analysis presented in this paper, we use one of the most common spatial econometric models proposed by the literature to include spatial effects [65, 66]: the spatial lag model. Mathematically, this model represents an extension of the baseline expression in Eq (2) that introduces a spatially autoregressive term as an additional right-hand side variable:

$$\begin{array}{c} LED=\alpha +\rho W\times LED+\beta LC+\gamma SP+\delta TX+\zeta ST+\epsilon \end{array}$$ (3)

where *W* is a *N* × *N* spatial weights matrix that encodes spatial relationships between the observations in the dataset (LSOA areas, in our case), *ρ* is the spatial autoregressive parameter, and the rest of the model is as in Eq (2). We use the queen contiguity criterion to build *W*, by which two areas are neighbors if they share any amount of border. We also row-standardize *W* so that the term *W* × *LED* –the so-called *spatial lag* of *LED*– represents the average LED index in the neighborhood of a given LSOA. Since the right-hand side of Eq (3) includes an endogenous variable, the model violates one of the assumptions on which OLS estimation rests –exogeneity– and hence rules the method out. Instead, the spatial econometric literature has proposed several alternatives [66]. We adopt a modern GMM [67–70] approach and use PySAL [71] to obtain estimates for the spatial lag model.

Predicted values of *LED* can be obtained using the reduced form of the model in Eq (3):

$$\begin{array}{c} L\hat{E}D={\left(I-\hat{\rho }W\right)}^{-1}\left(\hat{\alpha }+\hat{\beta }LC+\hat{\gamma }SP+\hat{\delta }TX+\hat{\zeta }ST\right) \end{array}$$ (4)

which does not require any value of *LED* to obtain a prediction from the fitted model. The expression above also serves to highlight a useful interpretation of the spatial lag model in this context: Akin to the time series equivalent, the model can be understood as a *spatial filter* that removes the effect of spatial autocorrelation that is ignored by the non-spatial counterpart in Eq (2).

### 0.5 Random Forest (RF)

We compare the traditional and spatial linear models above with two modern approaches to prediction that originated in the machine learning literature. Although there are several alternatives, we select the two we consider to be the most widely applied due to ease of use and overall performance. The first algorithm we introduce is Random Forest (RF, [72]). RF builds on other machine learning techniques, such as bagging (bootstrap aggregation), that aggregate a series of models into a single prediction. The key advantage of RF over bagging is the introduction of de-correlated trees, which significantly improves the variance of the prediction.

The algorithm underlying RF is composed of three main steps [73, Algorithm 15.1], and its intuition is rather straightforward. The first one consists in generating a series of subsets of the full dataset using bootstrap. For each of such subsets, the next step grows a decision tree using a random subsample of the variables in the dataset and produces a single outcome prediction. It is this bit, the random sampling of the variables considered in each of the trees, that produces much of the de-correlation between trees and provides RF with superior performance. In the third step, the predictions are averaged over all of the trees. Like decision trees, RF is able to capture non-linear relationships, does not suffer from numerical constraints relating to multicollinearity, and requires a minimal amount of manual tuning. Unlike single trees, the combination of several, decorrelated, models inherent to RF provides a significant improvement in its accuracy and predictive performance without sacrificing ease of use. A more in-depth description of the RF algorithm, its properties, and relative strengths and weaknesses is beyond the scope of this section. The interested reader is referred to [73] for an excellent description of the method.

Once fitted, RF can be exploited in ways that go beyond the simple use of their predictions. An additional device derived from RF (and other machine learning techniques such as gradient-boosted models; see below) is what is called “variable importance plot.” The main idea is to measure the extent to which each of the variables used in the RF contributes to improve predictions of the outcome variable. This is achieved by measuring improvement in a pre-defined criterion at each tree split where a variable is used. These improvements are then aggregated over the tree and averaged across the different trees that make up the forest. In this context, we use the residual sum of squares (RSS) as the importance criterion. That is, every time a variable is used for a split in a tree, we determine how much the RSS improves and then record this measure to construct the importance of that variable. Additionally, we can obtain a measure of the variability of these scores across trees in a forest.

### 0.6 Gradient Boost Regressor (GBR)

The second machine learning algorithm that is included is a Gradient Boost Regressor (GBR). Similar to the RF, it is an ensemble that combines the output of several models to produce a single prediction for the outcome variable. Boosting is a technique that improves the performance of an arbitrary predictive function, even if it produces weak predictions on its own [73, 74]. In this application, we use regression trees as the predicting function. Unlike RF, GBR operates in a sequential way: Each tree depends on results calculated in the previous step. As a result, the algorithm is not as parallelizable or scalable as the RF (where each tree can be run independently from the rest) but shows positive implications for its predictive performance and robustness.

The intuition of boosting algorithms is as follows: Using the entire dataset, fit the predicting function and obtain a set of estimates; plug them into a “loss function” to calculate the error of the model; use these errors (residuals), weighted with the previous ones, as the outcome to be predicted in the following iteration. Repeat this process a number of times to obtain several predictions. Average them using the same weights as those used for the adjustment. Given the simplicity of GBR, there are very few parts that require manual tuning, much like RF; thus, it is an almost automatic technique that is ideal for “off-the-shelf data mining applications” [73]. Additionally, the ability to specify different loss functions increases the robustness of the method to the effects of outliers.

GBR also allows the ability to derive tools that enhance the interpretability of the models estimated. In addition to importance plots, as with RF, we use in this context the so-called “partial dependence plot” (PDP). This graphical device illustrates the marginal relationship between a given (set of) variable(s) and the outcome or, in other words, its effect on the response once those of the other variables have been discounted [73, 74]. PDPs display, for a given value of the variable of interest, the average value of the predicting function combining such value with each of the values of the other variables. Although computationally intensive, these plots make it possible to identify not only whether a variable influences the outcome at all—positively, negatively, or in a non-linear way—but also to spot the values in which the effects are stronger.

## Results

We describe the main results according to the following precepts: model interpretation, to cover the output of each of the models estimated; and model performance, to assess in detail the relative advantages of each approach in predicting the LED index. Before we delve into the model output, let us first describe the choices we made when adapting each model to our dataset.

As described in Section, we derive a relatively large number of variables from the GE image. Although different in their own way, some of them, particularly those relating to spectral, texture and structure, are substantially correlated. This creates problems of multicollinearity when trying to fit all of them into a (spatial) regression model. For this reason, following similar studies (see [7] for an example of a similar approach), we use a dimensionality-reduction step to preserve as much of the variation contained in the entire set of variables while eliminating collinearity. We perform a principal components analysis on all the spectral, texture and structure variables and use the first four (rotated) factors as explanatory variables in the model. Those factors have an eigenvalue above 1 and account for 90% of the total variance. The rotated factor loadings are presented in Table 3; factor 1 (*f*1) captures all spectral variables and those variables from the texture group related to the standard deviation of the edgeness factor (DEVST_EDG), the covariance, variance and correlation of the GLCM, as well as the ratio variance at first lag (RVF) from the set of structure variables. Factor 2 (*f*2) captures a selection of texture and structure variables. The texture variables related to *f*2 indicate uniformity, roughness, entropy and contrast as well as SKEWNESS and KURTOSIS, which provide another way to characterize the homogeneity or heterogeneity of the urban layout [75]. The structure variables related to *f*2 (FDO, MFM, and DMF) provide information about the variability of image values: FDO indicates the variability changes at short distances, MFM informs about the global changes in the variability of the data, and DMF is directly related to the homogeneity of the values of the image [62]. Factors 3 (*f*3) and 4 (*f*4) summarize the remaining structure variables. Factor 3 captures the variables RSF, SDT, VFM, and RMM. According to [62], these structure variables convey information about the changes in variability and homogeneity at short distances (RSF and SDT respectively) and about the global homogeneity (VFM) and total variability (RMM) inside each LSOA. Factor 4 (*f*4) for the most part captures information on the structure variables SDF and AFM. SDF conveys information about homogeneity at short distances [62, 75]; it has been reported that its value increases as the homogeneity at short distances also increases for agricultural plots [63]; AFM provides another way to quantify the variability of the data [62].

**Table 3:** Rotated factor loadings (orthogonal Varimax rotation) of image spectral, texture and structure variables.

Variable	Factor 1	Factor 2	Factor 3	Factor 4
MEAN1	**0.8936**	0.346	0.002	-0.0633
DEVST1	**0.8366**	0.3036	0.3805	0.1993
MEAN2	**0.9475**	0.0755	-0.0178	0.0055
DEVST2	**0.8094**	0.3437	0.3938	0.2035
MEAN3	**0.903**	0.3533	0.0642	-0.0152
DEVST3	**0.849**	0.2793	0.3338	0.2287
MEAN_EDG	0.1691	**0.963**	-0.0661	0.0242
DEVST_EDG	**0.6485**	0.5929	0.0101	0.2671
UNIFOR	-0.2777	**-0.8503**	0.0135	-0.0167
ENTROP	0.4904	**0.8417**	0.0832	0.0861
CONTRAS	0.3685	**0.8944**	-0.0159	0.1263
IDM	-0.0082	**-0.951**	0.1062	0.0133
COVAR	**0.8522**	0.1433	0.4224	0.1904
VARIAN	**0.8435**	0.25	0.3923	0.1941
CORRELAC	**0.5757**	-0.5293	0.5393	0.2014
SKEWNESS	0.1689	**-0.6333**	0.1646	0.5480
KURTOSIS	-0.1572	**-0.6882**	-0.0055	0.4423
RVF	**0.6719**	-0.4935	0.467	0.0325
RSF	-0.1193	-0.308	**0.7009**	0.3285
FDO	0.3681	**0.8646**	0.1731	0.2296
SDT	0.2795	0.2227	**0.7764**	0.1445
MFM	0.5481	**0.7048**	0.2911	0.3145
VFM	0.3318	-0.078	**0.895**	0.0116
DMF	0.5817	**0.5939**	0.384	0.3641
RMM	0.3789	0.0888	**0.7737**	-0.3623
SDF	-0.4438	-0.5395	-0.0032	**-0.6643**
AFM	0.5871	0.2946	0.043	**0.6730**

Multicollinearity, on the contrary, is not a problem for tree-based methods. For this reason, we include all of the original spectral, texture and structure variables in the RF and GBR models. This allows the techniques to exploit all the variation and distill for themselves those that are relevant and useful from those that are not. Finally, although largely automatic when compared to similar approaches, the two machine learning methods we use require the tuning of a small number of parameters. One is the number of trees that compose the RF. A larger number potentially provides a more comprehensive and detailed pool of estimates that results in a more accurate final ensemble estimate; however, more trees also imply a greater computational burden. We grow both the RF and the GBR with 100 trees because, after several experiments using the mean squared error (MSE) as the performance metric, that number proved a good balance between accuracy and computational feasibility. Additionally, the GBR makes it possible to specify the loss function and the learning rate at which the residuals are updated in every iteration. We use the least absolute deviation loss function, following [73], who praise its robustness. For the learning rate, we adopt a data-driven approach and find the value that minimizes MSE using 5-fold cross-validation (see below for a description of the technique), a standard technique in the machine learning field.

### 0.7 Model interpretation

Interpretation of linear models is usually performed by examining the sign, size and significance of the estimated parameters. The main results for both the linear and spatial models are displayed in Table 4. The models include the four extracted factors—*f*1, *f*2, *f*3 and *f*4—as well as the percentages of the three land cover variables that prove most relevant: water (*p*_*b*_*water*), shadow (*p*_*shadow*), and vegetation (*p*_*veg*). In addition, the spatial model in columns 3-4 also includes the spatial lag of the dependent variable (*ρ*). Both the standard and the spatial model largely agree in the results: The third and fourth factors are significant, as are the proportion of an area occupied by water and vegetation. Neither the second factor nor the percentage of shadow seem to have significant explanatory power in predicting the level of LED. We also tested alternative specifications that include a few more explanatory variables, but we are not including them because they do not contribute fundamentally new insights, and we believe the interesting comparison in this paper is between linear models and machine learning approaches. Detailed results are, however, available from the authors.

**Table 4:** Regression coefficients.

	OLS		GMM		RF	GBR
	Coefficient	P-Value	Coefficient	P-Value		
CONSTANT	65.0581	0.0003	44.6907	0.0011		
f1	-1.7557	0.3315	-3.0328	0.0264		
f2	-2.6794	0.1764	-2.1430	0.1526		
f3	3.2969	0.0003	2.0437	0.0036		
f4	-2.0457	0.0209	-1.3078	0.0497		
p_b_water	-3.246	0.0000	-1.8888	0.0005		
p_shadow	-0.0983	0.7794	-0.3728	0.1599		
p_veg	-0.644	0.0075	-0.5402	0.0029		
*ρ*			0.6504	0.0000		
*R*^2^	0.3440		0.4327		0.9354	0.8320

Although results are comparable across models, some details do change when one introduces spatial effects. The significance of variables changes only for the first factor that becomes significant when the spatial effects are included. The size of the coefficients is smaller in the spatial model. These changes are a known effect of ignoring positive spatial autocorrelation when this is present and significant. In this case, some of the variation due to the spatial lag of the variable is picked up by other correlated explanatory variables, causing them to display inflated estimates. When the spatial effect is properly accounted for, as in the spatial lag model in this case, this variation is absorbed by the spatial lag term, and the other coefficients display a more conservative size. The presence of relevant spatial autocorrelation in our model is further confirmed by the significance and large size of the spatial parameter (*ρ*).

Regarding the signs of the significant variables, which do not change between the classical and the spatial model, larger proportions of water are associated with smaller deprivation, as is the share of vegetation. Smaller values of *f*1 and *f*4 and larger values of *f*3 indicate larger deprivation, characterized by urban layout patterns with high heterogeneity in short distances, high spatial regularity, few large homogeneous surfaces but high homogeneity at greater distances.

**Fig 2:**
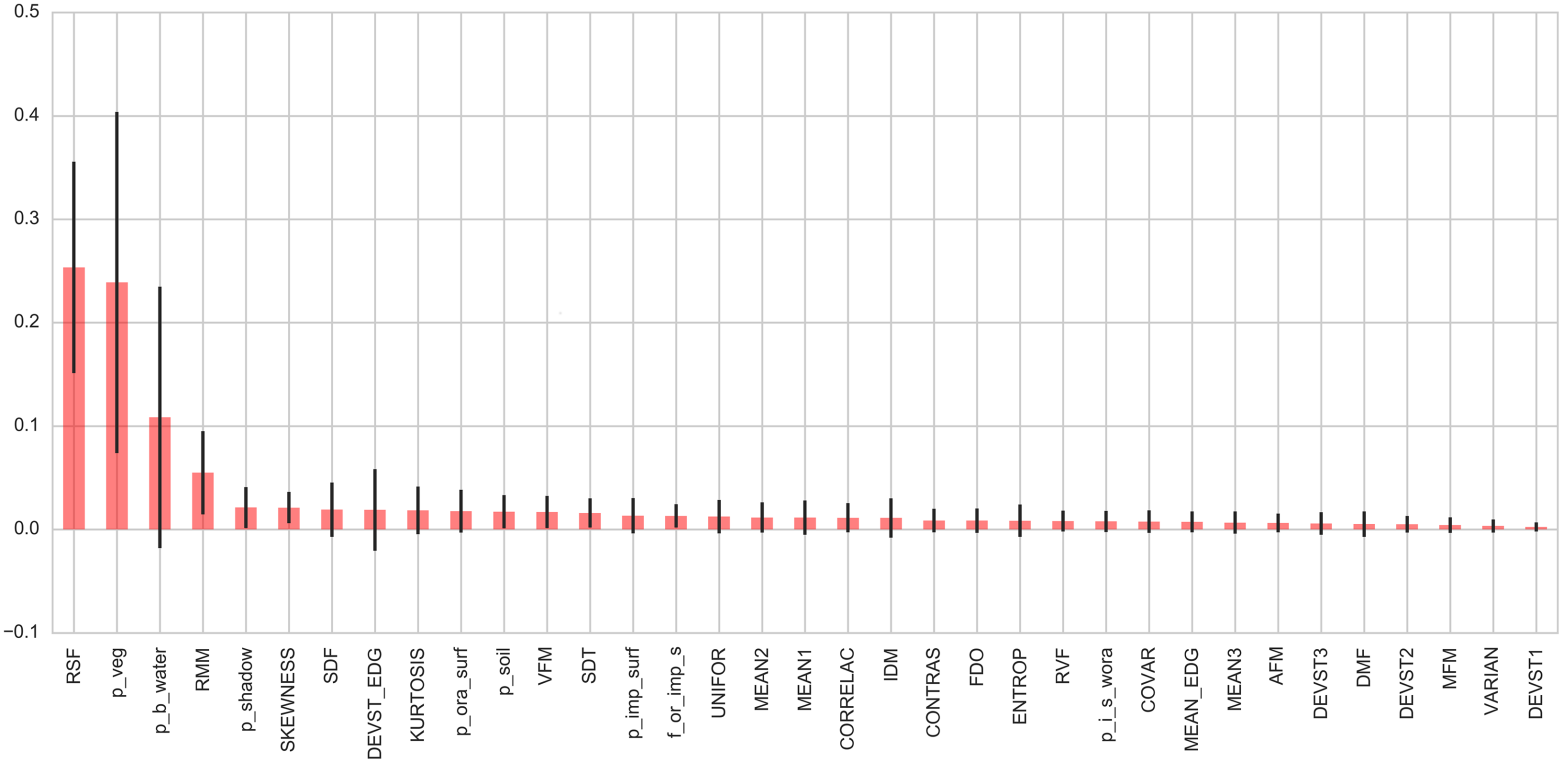
Feature importance plot (Random Forest).

Because of their non-parametric nature, neither the RF nor the GBR can be interpreted in similar terms as the models in Table 4. Instead, we use the feature importance plot and the PDP to gain insight into the relevance and shape of the relationships between explanatory variables and the response. Fig 2 displays the importance scores computed from the RF for each of the variables in the RF and GBR models (we only show the plot from RF because that from the GBR is equivalent and does not bring any additional insight). The chart shows that most of the predictive power is derived from just over four variables despite the fact that 35 were used. The two most relevant ones are *RSF*, a structure variable that informs about the changes in variability at short distances, and the share of vegetation (*p*_*veg*) in each area. These are followed by the share of water (*p*_*b*_*water*) and *RMM*, another structure variable that indicates total variability in each area. The black lines in each bar represent a measure of dispersion of the score across the trees that make up the forest. In some cases, these are substantial (e.g., the percentage of vegetation).

**Fig 3:**
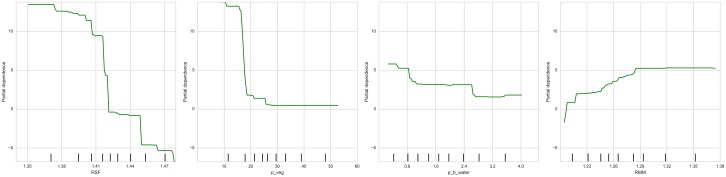
Partial dependence plots for the four most relevant variables (Gradient Boost Regressor): RSF, percentage of vegetation and water, and RMM.

To explore the relation between explanatory variables and the response, Fig 3 displays the PDPs derived from the GBR for the four most important variables: *RSF*, the percentages of vegetation and water, and *RMM*. On the horizontal axis is displayed the range of values the variable takes, while the vertical axis presents the values of the partial dependence function. The first three variables display an overall negative relationship with the response, while the fourth one is positively associated. There are substantial differences between each of them: The dependence function for *RSF* is almost flat until values around 1.39, when it drops sharply; the case for vegetation is the opposite, as most of the drop is concentrated in lower values, until 20%; the link between the share of water and LED is much more nuanced and concentrates in the lower and higher values; finally, RMM displays an almost positive linear relationship for lower values, until 1.29, where it flattens out for the rest of the values.

To some extent, it could be argued that part of this information is also provided by the regression model. The coefficients, significant in all cases, for both *p*_*veg* and *p*_*b*_*water* are negative and significant, and as obtained in ancillary regressions not shown, those for *RSF* and *RMM* are negative and positive, respectively (these regressions, which are available from the authors, included both variables instead of the factors obtained in the factor analysis). However, the variable importance and the partial dependence plots allow us to extend these views and exploit the additional flexibility and detail afforded by both the RF and GBR to obtain further insights.

### 0.8 Model performance

Once we have a good idea of how the models produce predictions; what the variables are, and which approach contributes most to generating the estimates of the LED index, we turn to the question of how good these predictions are. Validation and performance are important steps in any modeling exercise and, as such, have received considerable attention both in statistics (e.g., [76]) and machine learning (e.g., [73]). How easy they are to measure and compare depends on what the ultimate purpose of the exercise is. In our case, we are interested in obtaining the best possible estimates of the LED index using only data derived from satellite imagery. In other words, we want models that can produce predictions as accurately as possible, capturing as much proportion of the variation inherent in the data as possible. Ideally, we want our models to not only be able to explain the response variable they have *seen* but also other values that have not been presented to the model but are from the same generating process. There are several measures that can assist us in this evaluation, such as the mean squared error, or the *R*^2^. In the interest of comparing with other similar exercises in the literature (e.g., [7]), we opt for the coefficient of determination (*R*^2^), a popular index that measures the proportion of variance in the response that is captured by the explanatory set of variables. We also perform very similar comparisons using alternative measures such as (root) mean squared errors, and the results and conclusions were virtually the same. They are not included in the paper due to space constraints, but are available from the authors.

We approach the evaluation in two distinctive ways: One, we use the term “naive,” following the standard procedure in many of the social science applications of regression; and second, we use the term “honest” [77] based on cross-validation. In the naive assessment, we use the model output obtained based on the entire dataset and calculate the *R*^2^ for each model. This measures the ability of a model to capture the variation in data that the model already has *seen*; hence, the naive adjective. The scores for each model can be found in the bottom row of Table 4. There are two interesting elements that highlight the naive evaluation. First, it is the stark contrast between the (spatial) linear model performance and that of the machine learning techniques, which are substantially higher. In fact, it appears that using either RF or GBR, one can predict around 88% of the variation in LED using only data from satellites. This would point to a clear superiority of the latter over the former when it comes to predictive performance, but as seen below in the honest approach, there are factors that preclude a comparison in these terms. Second, focusing on the linear models, the inclusion of spatial effects in the form of a spatial lag results in a substantially improved performance, from around 34% of the variation explained to almost 44%.

However, our real interest in predictive performance usually relates to the ability of a model to generate good predictions not using the same data used to fit the model but using *new* data—observations that have not been used to fit the model in the first place. The gap between the two is usually referred to as overfitting, and it is a common but serious problem in predictive applications like the one we are undertaking. To avoid this situation, a typical approach is to split the data into two parts and then use one to *train* the model, reserving the other one to *test* its predictive performance. This is what, repeated a few times (folds), is called cross-validation (CV); it is a much more robust way of evaluating the ability of a model to predict responses.

**Fig 4:**
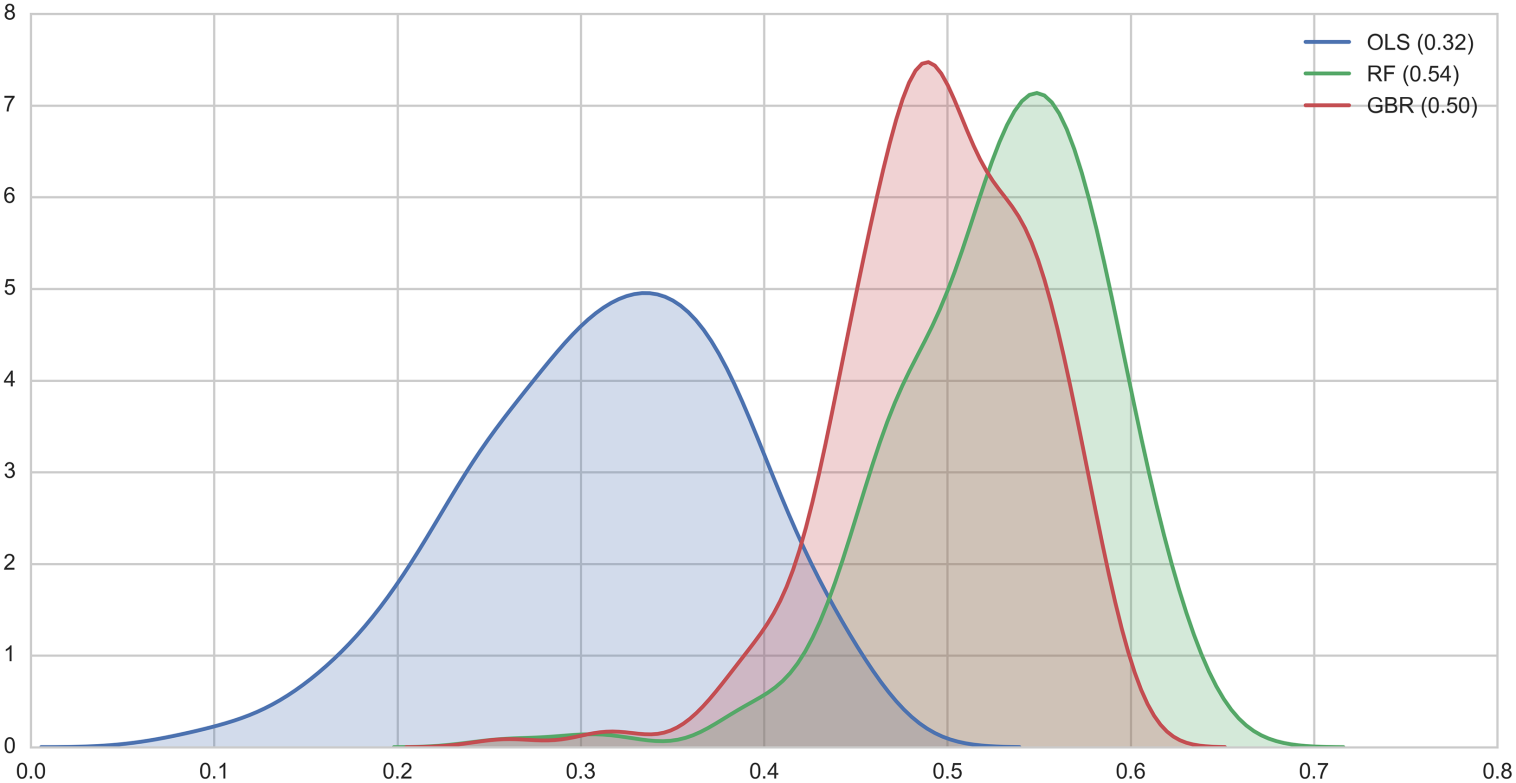
Cross-validated *R*^2^ (median values in parenthesis).

We adopt a CV approach to evaluate the non-spatial linear model and the two machine learning-based approaches, the RF and the GBR. In a spatial econometric model like the one in Eq (3), CV is not directly applicable because CV implicitly assumes the independence of each predicted value, while the spatial lag included in our model explicitly links observations through the channel of spatial connectivity. We obtain cross-validated scores for *R*^2^, repeating 250 times the split-train-test procedure in each iteration. This yields 250 scores for each of the three models. Fig 4 displays the density distribution of the scores for each of them. There are a few interesting aspects to highlight. First, the median values for each model are lower than their naive counterparts. This is particularly so in the case of the machine learning methods, which go from 0.94 (RF) and 0.83 (GBR) to 0.54 and 0.50, respectively. The linear model proves more robust to overfitting and only has a reduction of 0.04, from the naive 0.34 to 0.3 in the honest case. Second, the uncertainty behind the true *R*^2^ is smaller in the case of the RF and GBR as compared to that of the OLS linear model. This can be seen in the greater dispersion of the blue curve when observed next to the red and green ones. Third, and most importantly, even accounting for the reduction due to CV, our honest, CV-based estimates of the *R*^2^ for each model clearly show the superiority of the machine learning methods over the linear model when it comes to predicting the LED index in an area. Both of them can account for at least half of the variation in the response, and the RF, our best performing model, even has a median *R*^2^ of 0.54. This suggests that the flexibility of these techniques in incorporating complex, non-linear relationships in the data provides them with an added predictive bonus when compared to more traditional approaches such as linear models that rely on a pre-specified functional form. Finally, it is worth highlighting these levels or predictive performance are well in line with the literature, particularly the most recent one. To give a few points of comparison, [5] obtain *R*^2^ scores between 0.37 and 0.75 depending on the specific measure of poverty they are trying to predict and the country for which they obtain estimates; and [6] develop satellite-derived predictions of a welfare index and environmental metrics, obtaining accuracy levels between 31% and 61%.

## Discussion

The first key message delivered by the exercise on Liverpool just presented is the ability of satellite imagery to explain and successfully predict to a reasonable level of accuracy living environment deprivation. This result adds to a growing body of literature demonstrating the usefulness of these sources of data to predict several conceptualizations of human deprivation and urban quality of life (e.g., [5, 6, 10, 11, 23, 25, 78]).

We found two land cover variables with high predictive performance in all the tested models: the land cover variables percentage of vegetation (*p*_*veg*) and percentage of water (*p*_*b*_*water*). The negative relationship between the percentage of vegetation and the LED index can be understood as an evidence of the positive relationship between vegetation cover and socioeconomic conditions, which place our results in line with findings in other developed countries that show the explanatory power of vegetation as a predictor of socioeconomic conditions. Using different ways to quantify the presence of vegetation within an area, like the NDVI, the vegetation fraction or share of coverage, the tree density or the leaf area index, the results of previous works show positive and significant correlation between vegetation cover and socioeconomic conditions in the following cities: Montreal, Toronto, and Vancouver [41, 79]; Boston, Massachusetts [43]; Terre Haute, Indiana [29]; and Athens-Clarke County, Georgia [56]. However, [80] found almost the opposite: a significant and positive relationship between increases in urban vegetation and factors associated with urban decay in Detroit, Michigan. Other authors like [40], for the state of Massachusetts, and [42], for Philadelphia, Pennsylvania, found small areas in which this relationship is negative, inside a more general positive trend, providing evidence of spatial heterogeneities in the relationship between urban greenness and socioeconomic conditions.

Urban green spaces and water bodies can be considered as urban amenities that have a measurable impact in housing prices [49, 81–83], and also in urban quality of life [84]. The negative and significant relationship between *p*_*b*_*water* and LED is in agreement with previous works that relate the presence of water bodies to better urban quality of life [47, 48, 78]. Other land cover variables such as the share of impervious surfaces and clay roofs, which appear highly significant in cities like Medellin [7], do not show significant predictive power in our analysis. The share of impervious surfaces is highly and negatively correlated with the share of vegetation in Liverpool, hence the reduced complementary power of this feature when using OLS and spatial adjusted regressions along with the share of vegetation. However, the machine learning algorithms were able to capture the complementary effect of this variable to predict LED index values. On the other hand, the percentage of orange impervious surface cover, that indicates the presence of clay roofs, is a feature less indicative of wealth in developed countries because the presence of high rise expensive blocks that use new modern roofing types.

Texture and structure variables also proved to be useful for LED index estimation in Liverpool. Both sets of variables help characterize the spatial pattern of the urban layout [7, 75, 85]. Spatial pattern descriptors have been used to identify slums from remote sensing imagery in cities such as Campinas and Rio de Janeiro in Brazil [18, 86]; Cape Town, South Africa (Hofmann, 2001); Casablanca, Morocco [20]; Ahmedabad, Delhi, Pune and Hyderabad in India [21, 87–89]; and Medellin [7]. [23] reported that the most deprived areas in Accra are associated with less land cover variability. The most useful features from these sets of variables are RSF and RMM (Fig 2). Both features provide information of the spatial pattern by quantifying the changes in the variability of intensity values and of the variance at short distances [62]. In Liverpool, the areas with high RSF values tend to have large homogeneous green spaces and are associated with low LED index values, while the areas with high RMM values tend to have a periodic pattern of buildings in close proximity, few areas of large homogeneous spaces and are associated with high values of the LED index. Our results indicate that urban layout patterns in Liverpool with high heterogeneity in short distances, high spatial regularity, few large homogeneous surfaces and high homogeneity at larger distances are indicative of high levels of the LED index.

These results provide new evidence about the instability of the predictive variables for living conditions across countries. In particular, our findings stress the need to account for the particular conditions in each place when using remote sensing for place-based analysis [90] because findings for one city can be quite the opposite of those for another city [10, 17]. In context of this debate, it is worth highlighting an additional advantage of the machine learning approaches featured in this analysis. In contrast to the specific nature of the PCA results used in the linear models, both the RF and GBR provide the opportunity to include the raw measurements directly into the model, without previous transformations. This does not only make the modelling process more transparent and interpretable –as it is possible to assess the role each single variable plays– but also allows for easier and more direct comparisons with closely related literature. We anticipate that a greater adoption of this kind of methods will enhance the integration of similar studies and thus help build a better evidence base on how different contexts influence the predictive power of any given variable.

Finally, one of the advantages of using spatial regression is the ability to include contextual information and correlations that play out over space in the formal model. Given that the LED index is provided at the LSOA level, which is administratively defined and is likely not to follow the true underlying data generating process (DGP), the benchmark OLS estimates are likely to suffer from the well-known modifiable areal unit problem (MAUP, [64]). This is also true for the machine learning methods, which also take observations as isolated realizations of the DGP. This implies that since they cannot adequately capture the true underlying process, their predictions are not as accurate. The introduction of a spatial lag as in this study makes it possible to smooth part of the biases and breaks imposed by the administrative boundaries and thus adapt better to the true DGP, resulting in improved prediction quality. Our results confirm previous findings that the inclusion of explicitly spatial methodologies can improve results when the process observed has a spatial dimension, such as in the case of the LED index in Liverpool or, more generally, of other deprivation measures [7, 91–96]. Other ways to account for spatial dependence include designing analytical regions [97] that aggregate small areas into homogeneous and spatially contiguous regions, thus better reflecting the true underlying DGP. Analytical regions have been used by [23] and [7] in the context of poverty studies and have demonstrated good qualities for controlling spurious spatial autocorrelation and minimizing MAUP effects. In the case of both the RF and the GBR, this type of spatial problems are not as relevant of a problem as with the linear model because they do not impose any pre-defined structure on the DGP, so the trees grown by the classifiers are more flexible and adaptable.

## Conclusions

This paper explores the potential of two machine learning methods, GBR and RF, to predict the LED index in Liverpool (UK) using land cover, spectral, texture and structure variables extracted from a very high spatial resolution aerial image. We compare the results with the outcomes of two classic econometric models: OLS regression and a spatial lag model. In this model competition, RF proved to be the best model, with a median cross-validated *R*^2^ of 0.54, followed by GBR with 0.5, the spatial lag model with 0.43 (non-cross validated), and finally the OLS regression with 0.3.

Two structure variables, RSF and RMM, and two land cover variables, share of vegetation and share of water, show the best predictive power of the LED index in Liverpool. RSF indicates the changes in variability and homogeneity at short distances, and RMM indicates the total variability inside each LSOA polygon. In our case study, larger proportions of water and vegetation are associated with smaller deprivation. These results, when compared to previous studies, confirm that the variables that better explain socioeconomic indexes, like the Living Environment Deprivation, change between cities, regions and geographical contexts. More studies on this topic will make it possible to detect spatial patterns in the predictive power of image-extracted variables and, as a result, to determine the proper set of explanatory variables of living conditions of a city based on its geographical location.

In addition to noticeably better predictive performance, machine learning techniques, as illustrated by those used in this paper, offer a promising framework to include larger numbers of potential explanatory variables (features) derived from satellite images. This opens the door to the derivation of new variables for which, a priori, there is no substantive theory underlying their association with poverty or deprivation but might be found have a strong predictive power (this is the case, for example, of the recent work by [5]). In this sense, we firmly believe a broader embrace of this kind of methods will translate into more transparent and accurate predictions of measures of deprivation, even though these might come in part from variables without much substantive meaning in their socio-economic interpretation.

## References

[1] Jain S. Remote sensing application for property tax evaluation. International Journal of Applied Earth Observation and Geoinformation. 2008;10(1):109–121. 10.1016/j.jag.2007.10.008

[2] Taubenböck H, Wegmann M, Roth A, Mehl H, Dech S. Urbanization in India—Spatiotemporal analysis using remote sensing data. Computers, Environment and Urban Systems. 2009;33(3):179–188. 10.1016/j.compenvurbsys.2008.09.003

[3] Steele JE, Sundsøy PR, Pezzulo C, Alegana VA, Bird TJ, Blumenstock J, et al Mapping poverty using mobile phone and satellite data. J R Soc Interface. 2017;14(20160690). 10.1098/rsif.2016.0690 PMC5332562 28148765

[4] Grace K, Nagle NN, Husak G. Can Small-Scale Agricultural Production Improve Children’s Health? Examining Stunting Vulnerability among Very Young Children in Mali, West Africa. Annals of the American Association of Geographers. 2016;4452(March):1–16.

[5] Jean N, Burke M, Xie M, Davis WM, Lobell DB, Ermon S. Combining satellite imagery and machine learning to predict poverty. Science (New York, NY). 2016;353(6301):790–794. 10.1126/science.aaf7894 27540167

[6] Watmough GR, Atkinson PM, Saikia A, Hutton CW. Understanding the Evidence Base for Poverty-Environment Relationships using Remotely Sensed Satellite Data: An Example from Assam, India. World Development. 2016;78:188–203. 10.1016/j.worlddev.2015.10.031

[7] Duque JC, Patiño JE, Ruiz LA, Pardo-Pascual JE. Measuring intra-urban poverty using land cover and texture metrics derived from remote sensing data. Landscape and Urban Planning. 2015;135:11–21. 10.1016/j.landurbplan.2014.11.009

[8] Engstrom R, Sandborn A, Yu Q, Graesser J. Assessing the relationship between spatial features derived from high resolution satellite imagery and census variables in Accra, Ghana. IGARSS 2015. 2015; p. 2544–2547.

[9] Sandborn A, Engstrom RN. Determining the Relationship Between Census Data and Spatial Features Derived From High-Resolution Imagery in Accra, Ghana. IEEE Journal of Selected Topics in Applied Earth Observations and Remote Sensing. 2016;9(5):1970–1977. 10.1109/JSTARS.2016.2519843

[10] Patiño JE, Duque JC. A review of regional science applications of satellite remote sensing in urban settings. Computers, Environment and Urban Systems. 2013;37:1–17. 10.1016/j.compenvurbsys.2012.06.003

[11] Kuffer M, Pfeffer K, Sliuzas R. Slums from Space—15 Years of Slum Mapping Using Remote Sensing. Remote Sensing. 2016; 10.3390/rs8060455

[12] Jenerette GD, Harlan SL, Brazel A, Jones N, Larsen L, Stefanov WL. Regional relationships between surface temperature, vegetation, and human settlement in a rapidly urbanizing ecosystem. Landscape Ecology. 2007;22(3):353–365. 10.1007/s10980-006-9032-z

[13] Mennis J. Socioeconomic-vegetation relationships in urban, residential land: the case of Denver, Colorado. Photogrammetric Engineering and Remote Sensing. 2006;72(8):911–921. 10.14358/PERS.72.8.911

[14] Mennis J, Liu JW. Mining association rules in spatio-temporal data: An analysis of urban socioeconomic and land cover change. Transactions in GIS. 2005;9(1):5–17. 10.1111/j.1467-9671.2005.00202.x

[15] Rajasekar U, Weng Q. Application of association rule mining for exploring the relationship between urban land surface temperature and biophysical/social parameters. Photogrammetric Engineering and Remote Sensing. 2009;75(3):385–396. 10.14358/PERS.75.4.385

[16] Besussi E, Chin N, Batty M, Longley P. The structure and form of urban settlements In: Rashed T, Jürgens C, editors. Remote sensing of urban and suburban areas. Remote Sensing and Digital Image Processing. Dordrecht: Springer Netherlands; 2010 p. 13–31. Available from: http://www.springerlink.com/index/10.1007/978-1-4020-4385-7.

[17] de Sherbinin A, Balk D, Yager K, Jaiteh M, Pozzi F, Giri C, et al A CIESIN thematic guide to social science applications of remote sensing. Palisades, NY: Center for International Earth Science Information Network (CIESIN), Columbia University; 2002.

[18] Hofmann P, Strobl J, Blaschke T, Kux HJH. Detecting informal settlements from QuickBird data in Rio de Janeiro using an object-based approach In: Blaschke T, Lang S, Hay GJ, editors. Object-based image analysis—Spatial concepts for knowledge-driven remote sensing applications. New York: Springer; 2008 p. 531–553.

[19] Kohli D, Sliuzas R, Kerle N, Stein A. An ontology of slums for image-based classification. Computers, Environment and Urban Systems. 2012;36(2):154–163. 10.1016/j.compenvurbsys.2011.11.001

[20] Rhinane H, Hilali A, Berrada A, Hakdaoui M. Detecting slums from SPOT data in Casablanca Morocco using an object based approach. Journal of Geographic Information System. 2011;03(03):209–216. 10.4236/jgis.2011.33018

[21] Kit O, Lüdeke M, Reckien D. Texture-based identification of urban slums in Hyderabad, India using remote sensing data. Applied Geography. 2012;32(2):660–667. 10.1016/j.apgeog.2011.07.016

[22] Forster B. Some urban measurements from Landsat data. Photogrammetric Engineering and Remote Sensing. 1983;49(12):1693–1707.

[23] Weeks JR, Hill AG, Stow D, Getis A, Fugate D. Can we spot a neighborhood from the air? Defining neighborhood structure in Accra, Ghana. GeoJournal. 2007;69(1–2):9–22. 10.1007/s10708-007-9098-4 19478993 PMC2686612

[24] Stoler J, Daniels D, Weeks JR, Stow Da, Coulter LL, Finch BK. Assessing the utility of satellite imagery with differing spatial resolutions for deriving proxy measures of slum presence in Accra, Ghana. GIScience & Remote Sensing. 2012;49(1):31–52. 10.2747/1548-1603.49.1.31 23847453 PMC3705761

[25] Baud I, Kuffer M, Pfeffer K, Sliuzas R, Karuppannan S. Understanding heterogeneity in metropolitan India: The added value of remote sensing data for analyzing sub-standard residential areas. International Journal of Applied Earth Observation and Geoinformation. 2010;12(5):359–374. 10.1016/j.jag.2010.04.008

[26] Rashed T. Sustainable hazards mitigation In: Campagna M, editor. GIS for sustainable development: Bringing geographic information science into practice towards sustainability. New York: Taylor & Francis (CRC Press); 2005 p. 287–309.

[27] Chen J, Chen S. Mental health effects of perceived living environment and neighborhood safety in urbanizing China. Habitat International. 2015;46:101–110. 10.1016/j.habitatint.2014.11.002

[28] Zainal NR, Kaur G, Ahmad NA, Khalili JM. Housing Conditions and Quality of Life of the Urban Poor in Malaysia. Procedia—Social and Behavioral Sciences. 2012;50(July 2012):827–838. 10.1016/j.sbspro.2012.08.085

[29] Jensen R, Gatrell J, Boulton J, Harper B. Using remote sensing and geographic information systems to study urban quality of life and urban forest amenities. Ecology and Society. 2004;9(5):5–15. 10.5751/ES-01201-090505

[30] Lo CP, Faber BJ. Integration of Landsat Thematic Mapper and census data for quality of life assessment. Remote Sensing of Environment. 1997;62(2):143–157. 10.1016/S0034-4257(97)00088-6

[31] Weber C, Hirsch J. Some urban measurements from SPOT data: urban life quality indices. International Journal of Remote Sensing. 1992;13(17):3251–3261. 10.1080/01431169208904116

[32] Smith T, Noble M, Noble S, Wright G, McLennan D, Plunkett E. The English Indices of Deprivation 2015: Technical Report. London: Department of Communities and Local Government; 2015. September. Available from: https://www.gov.uk/government/uploads/system/uploads/attachment_data/file/464485/English_Indices_of_Deprivation_2015_-_Technical-Report.pdf.

[33] Owen KK, Wong DW. An approach to differentiate informal settlements using spectral, texture, geomorphology and road accessibility metrics. Applied Geography. 2013;38:107–118. 10.1016/j.apgeog.2012.11.016

[34] Taubenböck H, Kraff NJ. The physical face of slums: a structural comparison of slums in Mumbai, India, based on remotely sensed data. Journal of Housing and the Built Environment. 2014;29(1):15–38. 10.1007/s10901-013-9333-x

[35] Kohli D, Sliuzas R, Stein A. Urban slum detection using texture and spatial metrics derived from satellite imagery. Journal of Spatial Science. 2016;8596(May):1–22.

[36] Yang X, Jiang GM, Luo X, Zheng Z. Preliminary mapping of high-resolution rural population distribution based on imagery from Google Earth: A case study in the Lake Tai basin, eastern China. Applied Geography. 2012;32(2):221–227. 10.1016/j.apgeog.2011.05.008

[37] Hu Q, Wu W, Xia T, Yu Q, Yang P, Li Z, et al Exploring the use of Google Earth imagery and object-based methods in land use/cover mapping. Remote Sensing. 2013;5(11):6026–6042. 10.3390/rs5116026

[38] Dell’Acqua F, Stasolla M, Gamba P. Unstructured human settlement mapping with SAR sensors. Proc of IGARSS06. 2006;30.

[39] Huang X, Liu H, Zhang L. Spatiotemporal Detection and Analysis of Urban Villages in Mega City Regions of China Using High-Resolution Remotely Sensed Imagery. IEEE Transactions on Geoscience and Remote Sensing. 2015;53(7):3639–3657. 10.1109/TGRS.2014.2380779

[40] Ogneva-Himmelberger Y, Pearsall H, Rakshit R. Concrete evidence & geographically weighted regression: A regional analysis of wealth and the land cover in Massachusetts. Applied Geography. 2009;29(4):478–487. 10.1016/j.apgeog.2009.03.001

[41] Tooke TR, Klinkenberg B, Coops NC. A geographical approach to identifying vegetation-related environmental equity in Canadian cities. Environment and Planning B: Planning and Design. 2010;37(6):1040–1056. 10.1068/b36044

[42] Pearsall H, Christman Z. Tree-lined lanes or vacant lots? Evaluating non-stationarity between urban greenness and socio-economic conditions in Philadelphia, Pennsylvania, USA at multiple scales. Applied Geography. 2012;35(1–2):257–264. 10.1016/j.apgeog.2012.07.006

[43] Duncan DT, Kawachi I, Kum S, Aldstadt J, Piras G, Matthews SA, et al A Spatially explicit approach to the stydy of socio-demographic inequality in the spatial distribution of trees across Boston neighborhoods. Spatial Demography. 2014;2(1):1–29. 10.1007/BF03354902 29354668 PMC5771436

[44] Stokes P. 2011 Census: Characteristics of Built-Up Areas; 2013.

[45] Sykes O, Brown J, Cocks M, Shaw D, Couch C. A city profile of Liverpool. Cities. 2013;35:299–318. 10.1016/j.cities.2013.03.013

[46] Office of National Statistics. Census geography—An overview of the various geographies used in the production of statistics collected via the UK census; 2011.

[47] Panagopoulos T, González Duque JA, Bostenaru Dan M. Urban planning with respect to environmental quality and human well-being. Environmental Pollution. 2016;208:137–144. 10.1016/j.envpol.2015.07.038 26243477

[48] Serag El Din H, Shalaby A, Farouh HE, Elariane SA. Principles of urban quality of life for a neighborhood. HBRC Journal. 2013;9(1):86–92. 10.1016/j.hbrcj.2013.02.007

[49] Li X, Zhang C, Li W, Ricard R, Meng Q, Zhang W. Assessing street-level urban greenery using Google Street View and a modified green view index. Urban Forestry and Urban Greening. 2015;14(3):675–685. 10.1016/j.ufug.2015.06.006

[50] Gupta K, Kumar P, Pathan SK, Sharma KP. Urban Neighborhood Green Index—A measure of green spaces in urban areas. Landscape and Urban Planning. 2012;105(3):325–335. 10.1016/j.landurbplan.2012.01.003

[51] Cabrera-Barona P, Wei C, Hagenlocher M. Multiscale evaluation of an urban deprivation index: Implications for quality of life and healthcare accessibility planning. Applied Geography. 2016;70:1–10. 10.1016/j.apgeog.2016.02.009

[52] Jorgensen A, Gobster PH. Shades of Green: Measuring the Ecology of Urban Green Space in the Context of Human Health and Well-Being. Nature and Culture. 2010;5(3):338–363. 10.3167/nc.2010.050307

[53] Wolch JR, Byrne J, Newell JP. Urban green space, public health, and environmental justice: The challenge of making cities ‘just green enough’. Landscape and Urban Planning. 2014;125:234–244. 10.1016/j.landurbplan.2014.01.017

[54] Weng Q, Xu B, Hu X, Liu H. Use of earth observation data for applications in public health. Geocarto International. 2013;29(1):3–16. 10.1080/10106049.2013.838311

[55] Kuffer M, Pfeffer K, Sliuzas R, Baud I. Extraction of Slum Areas From VHR Imagery Using GLCM Variance. IEEE Journal of Selected Topics in Applied Earth Observations and Remote Sensing. 2016;9(5):1830–1840. 10.1109/JSTARS.2016.2538563

[56] Lo CP. Application of LandSat TM data for quality of life assessment in an urban environment. Computers, Environment and Urban Systems. 1997;21(3–4):259–276. 10.1016/S0198-9715(97)01002-8

[57] Li G, Weng Q. Measuring the quality of life in city of Indianapolis by integration of remote sensing and census data. International Journal of Remote Sensing. 2007;28(2):249–267. 10.1080/01431160600735624

[58] Ruiz LA, Recio JA, Fernández-Sarría A, Hermosilla T. A feature extraction software tool for agricultural object-based image analysis. Computers and Electronics in Agriculture. 2011;76(2):284–296. 10.1016/j.compag.2011.02.007

[59] Janhäll S. Review on urban vegetation and particle air pollution—Deposition and dispersion. Atmospheric Environment. 2015;105:130–137. 10.1016/j.atmosenv.2015.01.052

[60] Barros M. Slums detection through lacunarity-based texture analysis of remote sensing images In: Expert group meeting on slum mapping. ITC, The Netherlands. Enschede: ITC, UN-HABITAT, CIESIN; 2008 p. 1–38.

[61] Haralick RM, Shanmugam K, Dinstein I. Textural features for image classification. IEEE Transactions on Systems, Man, and Cybernetics,. 1973;SMC-3(6):610–621. 10.1109/TSMC.1973.4309314

[62] Balaguer A, Ruiz LA, Hermosilla T, Recio JA. Definition of a comprehensive set of texture semivariogram features and their evaluation for object-oriented image classification. Computers & Geosciences. 2010;36(2):231–240. 10.1016/j.cageo.2009.05.003

[63] Balaguer-Beser A, Ruiz LA, Hermosilla T, Recio JA. Using semivariogram indices to analyse heterogeneity in spatial patterns in remotely sensed images. Computers and Geosciences. 2013;50:115–127. 10.1016/j.cageo.2012.08.001

[64] Openshaw S. The modifiable areal unit problem, CATMOG 38. Vol. 38 ed. Norwich: Geo Books; 1984 Available from: http://qmrg.org.uk/files/2008/11/38-maup-openshaw.pdf.

[65] Anselin L. Spatial econometrics: Methods and models. Dordrecht: Kluwer Academic Publishers; 1988.

[66] Anselin L, Rey SJ. Modern spatial econometrics in practice: A guide to GeoDa, GeoDaSpace and PySAL. GeoDa Press; 2014.

[67] Kelejian HH, Prucha IR. A generalized spatial two-stage least squares procedure for estimating a spatial autoregressive model with autoregressive disturbances. The Journal of Real Estate Finance and Economics. 1998;17(1):99–121. 10.1023/A:1007707430416

[68] Kelejian HH, Prucha IR. A Generalized Moments Estimator for the Autoregressive Parameter in a Spatial Model. International Economic Review. 1999;40(2):509–533. 10.1111/1468-2354.00027

[69] Kelejian HH, Prucha IR. Specification and estimation of spatial autoregressive models with autoregressive and heteroskedastic disturbances. Journal of Econometrics. 2010 10.1016/j.jeconom.2009.10.025 20577573 PMC2888178

[70] Arraiz I, Drukker DM, Kelejian HH, Prucha IR. A Spatial Cliff-Ord-type Model with Heteroskedastic Innovations: Small and Large Sample Results. Journal of Regional Science. 2010;50(2):592–614. 10.1111/j.1467-9787.2009.00618.x

[71] Rey SJ, Anselin L. PySAL: A Python library of spatial analytical methods In: Handbook of applied spatial analysis. Springer; 2010 p. 175–193.

[72] Breiman L. Random forests. Machine learning. 2001;45(1):5–32. 10.1023/A:1010933404324

[73] Friedman J, Hastie T, Tibshirani R. The elements of statistical learning. vol. 1 Springer series in statistics Springer, Berlin; 2001.

[74] James G, Witten D, Hastie T, Tibshirani R. An introduction to statistical learning. vol. 6 Springer; 2013.

[75] Duque JC, Patino JE, Ruiz LA, Pardo-Pascual JE. Quantifying slumness with remote sensing data. CIEF working paper, EAFIT University, No. 13-23; 2013.

[76] Gelman A, Hill J. Data analysis using regression and multilevel/hierarchical models. Cambridge University Press; 2006.

[77] Athey S, Imbens G. Recursive partitioning for heterogeneous causal effects. Proceedings of the National Academy of Sciences. 2016;113(27):7353–7360. 10.1073/pnas.1510489113 PMC4941430 27382149

[78] Tapiador FJ, Avelar S, Tavares-Corrêa C, Zah R. Deriving fine-scale socioeconomic information of urban areas using very high-resolution satellite imagery. International Journal of Remote Sensing. 2011;33(21):6437–6456. 10.1080/01431161.2010.512928

[79] Carrier M, Apparicio P, Kestens Y, Séguin AM, Pham H, Crouse D, et al Application of a Global Environmental Equity Index in Montreal: Diagnostic and Further Implications. Annals of the American Association of Geographers. 2016;1066(January 2017):1268–1285. 10.1080/24694452.2016.1197766

[80] Emmanuel R. Urban vegetational change as an indicator of demographic trends in cities: the case of Detroit. Environment and Planning B: Planning and Design. 1997;24(3):415–426. 10.1068/b240415 12293063

[81] Cho SH, Bowker JM, Park WM. Measuring the contribution of water and green space amenities to housing values: An application and comparison of spatially weighted hedonic models. Journal of Agricultural and Resource Economics. 2006;31(3):485–507. http://jareonline.org.

[82] Noonan DS. Finding an Impact of Preservation Policies: Price Effects of Historic Landmarks on Attached Homes in Chicago, 1990–1999. Economic Development Quarterly. 2007;21(1):17–33. 10.1177/0891242406296326

[83] Hu S, Yang S, Li W, Zhang C, Xu F. Spatially non-stationary relationships between urban residential land price and impact factors in Wuhan city, China. Applied Geography. 2016;68:48–56. 10.1016/j.apgeog.2016.01.006

[84] Nilsson P. Natural amenities in urban space—A geographically weighted regression approach. Landscape and Urban Planning. 2014;121:45–54. 10.1016/j.landurbplan.2013.08.017

[85] Patino JE, Duque JC, Pardo-Pascual JE, Ruiz LA. Using remote sensing to assess the relationship between crime and the urban layout. Applied Geography. 2014;55:48–60. 10.1016/j.apgeog.2014.08.016

[86] Barros M, Sobreira F. Analysing spatial patterns in slums: A multiscale approach In: Congresso Planejamento Urbano Regional Integrado Sustentável—PLURIS. Sao Carlos; 2005 p. 1.

[87] Niebergall S, Loew A, Mauser W. Object-oriented analysis of very high-resolution QuickBird data for mega city research in Delhi, India In: IEEE, editor. 2007 Urban Remote Sensing Joint Event. Paris, France: IEEE; 2007 p. 0–7.

[88] Shekhar S. Detecting slums from Quick Bird data in Pune using an object oriented approach. International Archives of the Photogrammetry, Remote Sensing and Spatial Information Sciences. 2012;XXXIX(B8):519–524. 10.5194/isprsarchives-XXXIX-B8-519-2012

[89] Kohli D, Warwadekar P, Kerle N, Sliuzas R, Stein A. Transferability of Object-Oriented Image Analysis Methods for Slum Identification. Remote Sensing. 2013;5(9):4209–4228. 10.3390/rs5094209

[90] Rashed T, Weeks J, Couclelis H, Herold M. An integrative GIS and remote sensing model for place-based urban vulnerability analysis In: Mesev V, editor. Integration of GIS and remote sensing. John Wiley & Sons, Ltd; 2007 p. 199–233.

[91] Duncan DT, Aldstadt J, Whalen J, White K, Castro MC, Williams DR. Space, race, and poverty: Spatial inequalities in walkable neighborhood amenities? Demographic Research. 2012;26(17):409–448. 10.4054/DemRes.2012.26.17 29046612 PMC5642981

[92] Sowunmi FA, Akinyosoye VO, Okoruwa VO, Omonona BT. The Landscape of Poverty in Nigeria: A Spatial Analysis Using Senatorial Districts- level Data. American Journal of Economics. 2012;2(5):61–74. 10.5923/j.economics.20120205.01

[93] Holt JB. The topography of poverty in the United States: a spatial analysis using county-level data from the Community Health Status Indicators project. Preventing chronic disease. 2007;4(4):1–9. PMC2099276 17875255

[94] Okwi PO, Ndeng’e G, Kristjanson P, Arunga M, Notenbaert A, Omolo A, et al Spatial determinants of poverty in rural Kenya. Proceedings of the National Academy of Sciences of the United States of America. 2007;104(43):16769–16774. 10.1073/pnas.0611107104 17942704 PMC2040447

[95] Voss PR, Long DD, Hammer RB, Friedman S. County child poverty rates in the US: a spatial regression approach. Population Research and Policy Review. 2006;25(4):369–391. 10.1007/s11113-006-9007-4

[96] Orford S. Identifying and comparing changes in the spatial concentrations of urban poverty and affluence: a case study of inner London. Computers, Environment and Urban Systems. 2004;28(6):701–717. 10.1016/j.compenvurbsys.2003.07.003

[97] Duque JC, Anselin L, Rey SJ. The max-p-regions problem. Journal of Regional Science. 2012;52(3):397–419. 10.1111/j.1467-9787.2011.00743.x

